# Recognition of Handwriting from Electromyography

**DOI:** 10.1371/journal.pone.0006791

**Published:** 2009-08-26

**Authors:** Michael Linderman, Mikhail A. Lebedev, Joseph S. Erlichman

**Affiliations:** 1 Norconnect Inc., Canton, New York, United States of America; 2 Department of Neurobiology, Duke University, Durham, North Carolina, United States of America; 3 Department of Biology, St. Lawrence University, Canton, New York, United States of America; Sun Yat-Sen University, China

## Abstract

Handwriting – one of the most important developments in human culture – is also a methodological tool in several scientific disciplines, most importantly handwriting recognition methods, graphology and medical diagnostics. Previous studies have relied largely on the analyses of handwritten traces or kinematic analysis of handwriting; whereas electromyographic (EMG) signals associated with handwriting have received little attention. Here we show for the first time, a method in which EMG signals generated by hand and forearm muscles during handwriting activity are reliably translated into both algorithm-generated handwriting traces and font characters using decoding algorithms. Our results demonstrate the feasibility of recreating handwriting solely from EMG signals – the finding that can be utilized in computer peripherals and myoelectric prosthetic devices. Moreover, this approach may provide a rapid and sensitive method for diagnosing a variety of neurogenerative diseases before other symptoms become clear.

## Introduction

The development of systems that can interface bioelectric activity to external devices hold significant clinical promise. For example, neural prosthetics strive to restore limb mobility and communication capacity in disabled subjects by interfacing brain potentials [1], [2], [3] or EMG activity [4], [5], [6] to artificial actuators or devices based on functional electrical stimulation [7], [8]. In addition to clinical applications, it has been suggested that bioelectric interfaces may even be used to enhance certain functions in normal subjects [9]. Although handwriting is one of the most important motor activities, it has received little attention from the designers of bioelectric interfaces due to perceived technical limitations and the paucity of models [10]. An interface that converts human bioelectric activity into text records could have a number of broad applications. First the development of this technology could substitute for computer peripherals or touch screens which have typically been used to record and transmit text messages. Bioelectric interfaces potentially could extract normal handwriting patterns directly from hand and arm EMGs. Clinically, handwriting features have been used for diagnostic purposes for patients with Parkinson's disease [11] and more recently dysgraphia has been shown to be a conserved element in the progression of Alzheimer's disease [12]. Methods that could be used to model handwriting could be used to diagnose diseases with a graphomotor component or be used to grade the progression of the disease or treatment.

**Figure 1:**
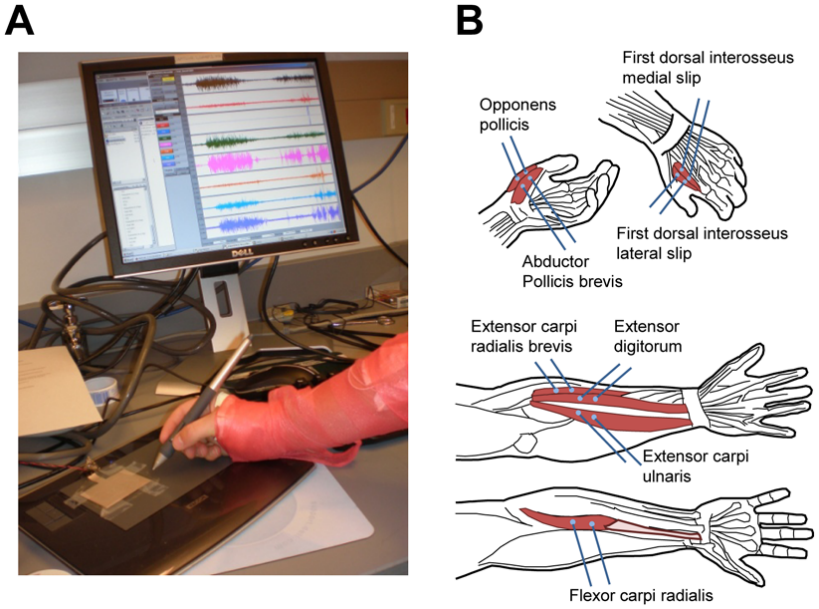
Data acquisition. A: A photograph of a recording session. B: Electrode placement over the hand (top) and forearm muscles (bottom).

**Figure 2:**
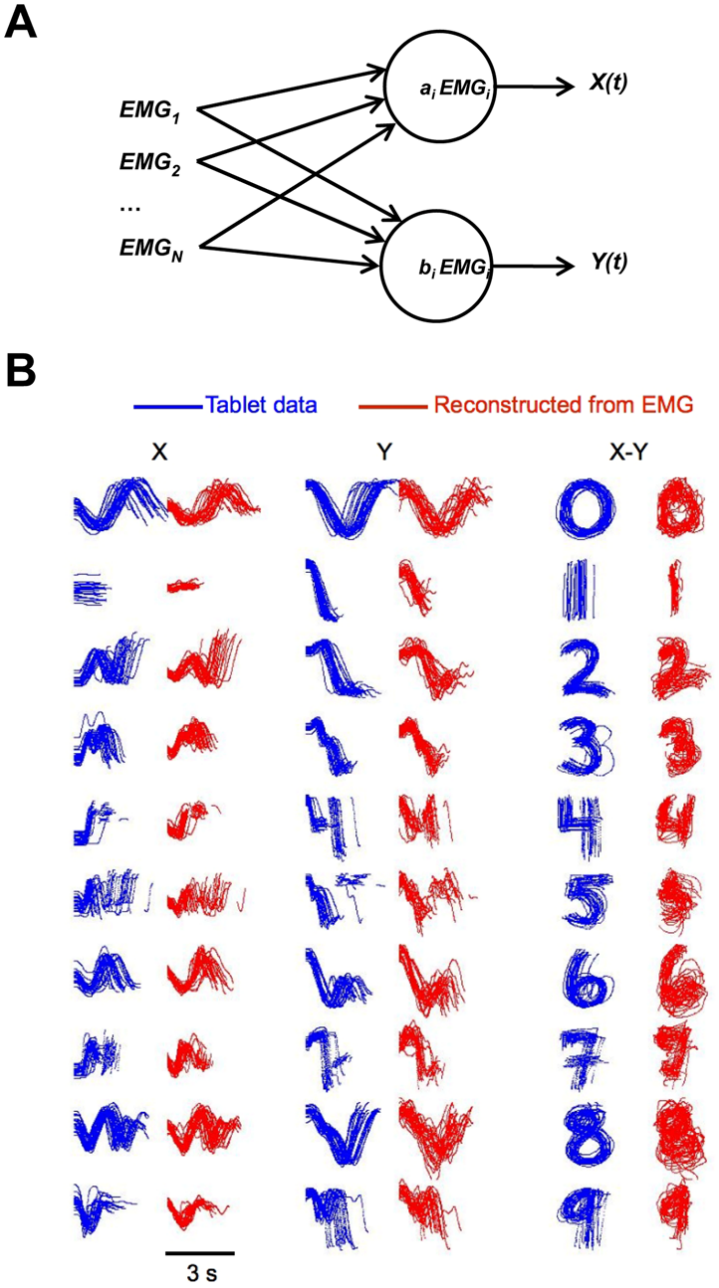
Reconstruction of handwriting traces using the Wiener filter. A: Schematics of the Wiener filter. EMG signals (rectified EMGs) from multiple models were fed into two independent Wiener filters which reconstructed *X* and *Y* coordinates of the pen, respectively. Each filter represented reconstructed coordinate as a weighted sum of EMGs. B: Examples of reconstructed traces from one recording session. Actual traces are shown in blue; reconstructed traces are shown in red. The first two columns show *X(t)* and *Y(t)*, respectively. The third column shows *X-Y* plots.

**Figure 3:**
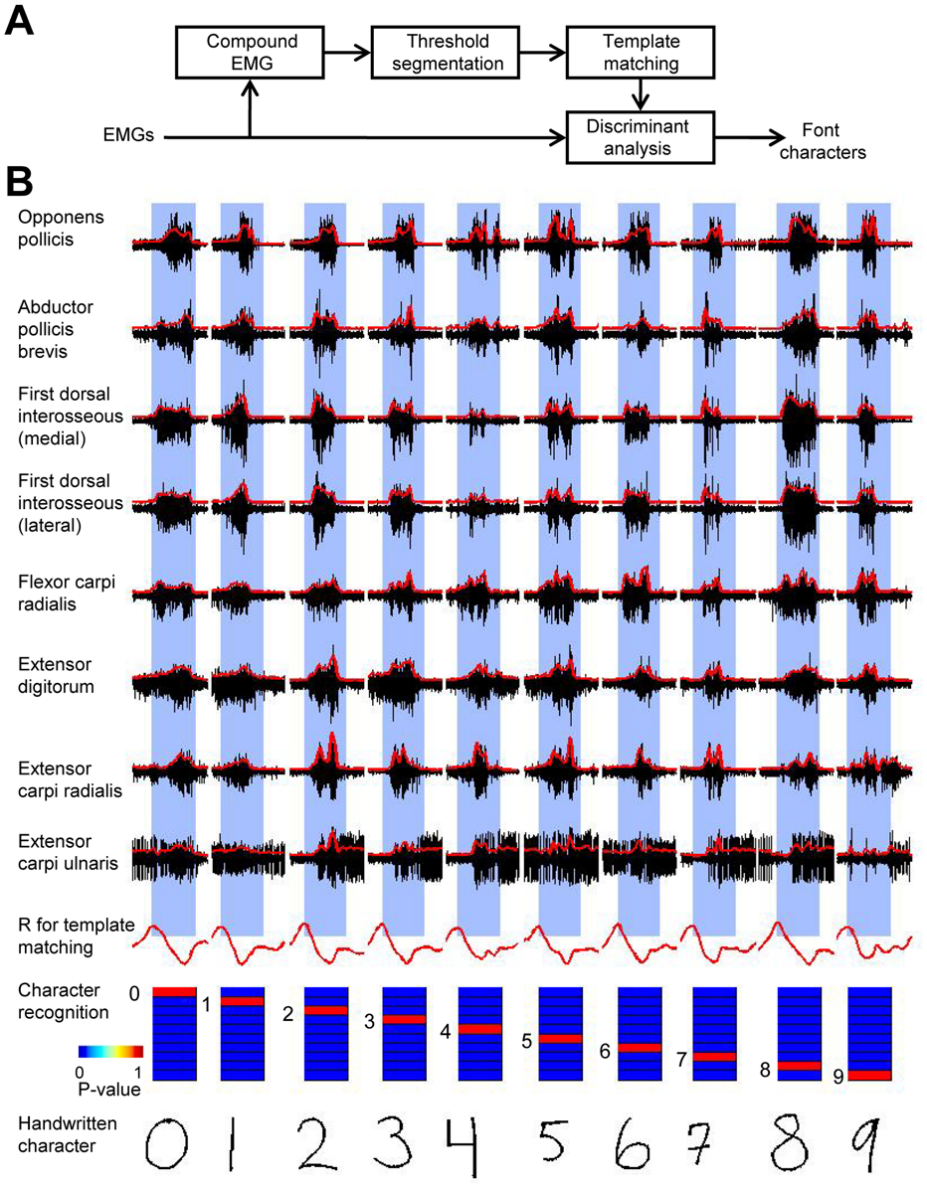
Transformation of EMG records into font characters. A: Schematics of the algorithm. Compound EMG (the sum of all rectified EMGs) was first used to detect the periods during which handwriting occurred. Compound EMG was first segmented into epochs corresponding to individual characters using a threshold that detected EMG bursts. Then, a generic compound EMG template was calculated by averaging these epochs. Template matching was used to refine the EMG segments, which were then classified using linear discriminant analysis. B: Example of discrimination for a representative recording session. From top to bottom: Eight EMGs were used for character recognition. 3.5-s segments corresponding to individual characters are highlighted as blue bars which are aligned on peak correlation coefficient, *R*, for template matching. Posterior probabilities for character recognition which were computed by discriminant analysis are shown as color plots. Recognized font character which corresponds to the highest probability is shown near each plot. Original handwriting is shown at the bottom.

The goal of this study was to develop a hardware/software system to record bioelectrical signals from the forearm and hand muscles (Fig. 1) and decode these signals with algorithms to extract and reproduce handwritten characters (Figs. 2 and 3).

## Results

We implemented two fundamental approaches for decoding handwriting from the EMGs. In the first approach, we *reconstructed* pen traces using linear decoding algorithm, the Wiener filter [13], [14] (Fig. 2). In the second approach, we *recognized* handwritten characters from the EMG patterns and displayed them as textual fonts. Thus, EMG patterns were mapped to discrete font characters. Both the reconstruction algorithms and the recognition algorithms had to be trained on the data from individual subjects and did not generalize to other subjects because of inter-subject variability.

As shown in Fig. 1B, bipolar surface EMG electrodes were placed on the skin overlying four forearm muscles and four hand muscles. Each of the muscles recorded exhibited EMG bursts during handwriting (Fig. 1A). Following conventional methodology [15], the intensity of EMG modulations was quantified as rectified EMG. To reconstruct the pen trace, the Wiener filters expressed *X* (left-right dimension) and *Y* (bottom-top) coordinates of the pen with respect to the writing surface as weighted sums of the rectified EMGs (Fig. 2A). The results of such reconstruction are shown in Fig. 2B. Pen traces recorded by the digitizing tablet are shown in blue, and the traces reconstructed from the EMGs are shown in red. The reconstructed traces followed the original handwriting with accuracy comparable to other bioelectrical interfaces [2].

The quality of reconstruction was evaluated as coefficient of determination, *R^2^*. *R^2^* values for individual subjects and statistics for the whole group are presented in Table 1. For the 6 subjects involved in these experiments, *R^2^* was 0.47±0.20 (mean±standard deviation across subjects) for *X* and 0.63±0.15 for *Y*. (*R^2^* can range from 0 to 1, and it reflects the proportion of variance in the original data captured by the reconstruction.) Table 1 also shows *R^2^* values for hand and forearm muscles. When only hand-muscle recordings were used for the reconstruction, *R^2^* was 0.26±0.10 for *X* and 0.50±0.12 for *Y*. When only forearm-muscle recordings were used, *R^2^* was 0.43±0.21 for *X* and 0.51±0.13 for *Y*. Pen position could be reconstructed even from EMGs of single muscles, although the accuracy was less compared to multiple-muscle reconstructions (Table 2). When the best reconstructing muscle was selected, *R^2^* was 0.31±0.17 for *X* and 0.32±0.10 for *Y* (Table 1).

**Table 1:** Reconstruction and recognition accuracy for individual subjects, combinations of recorded muscles and across-subject averages.

Subject	1	2	3	4	5	6	mean±st. dev.
Reconstruction,	R^2^						
All 8 EMGs	X: 0.72	0.19	0.54	0.44	0.62	0.31	0.47±0.20
	Y: 0.77	0.40	0.71	0.66	0.76	0.50	0.63±0.15
4 hand EMGs	X: 0.38	0.13	0.23	0.27	0.36	0.18	0.26±0.10
	Y: 0.69	0.35	0.58	0.49	0.48	0.42	0.50±0.12
4 forearm EMGs	X: 0.71	0.15	0.51	0.37	0.57	0.25	0.43±0.21
	Y: 0.52	0.29	0.55	0.58	0.67	0.42	0.51±0.13
1 best – all	X: 0.52 (#8)	0.11 (#7)	0.38 (#7)	0.22 (#7)	0.49 (#7)	0.17 (#7)	0.31±0.17
	Y: 0.57 (#1)	0.22 (#3)	0.42 (#1)	0.45 (#7)	0.35 (#7)	0.32 (#1)	0.39±0.12
1 best - hand	X: 0.27 (#4)	0.06 (#1)	0.09 (#4)	0.13 (#3)	0.28 (#4)	0.09 (#4)	0.15±0.10
	Y: 0.57 (#1)	0.22 (#3)	0.42 (#1)	0.35 (#3)	0.18 (#4)	0.32 (#1)	0.34±0.14
1 best - forearm	X: 0.52 (#8)	0.11 (#7)	0.38 (#7)	0.22 (#7)	0.49 (#7)	0.17 (#7)	0.31±0.17
	Y: 0.31 (#5)	0.14 (#8)	0.36 (#5)	0.45 (#7)	0.35 (#7)	0.32 (#8)	0.32±0.10
Recognition,	% correct						
All 8 EMGs	97.5	81.8	97.1	92.4	82.0	91.5	90.4±7.0
4 hand EMGs	87.3	69.7	89.6	84.3	62.8	81.3	79.2±10.6
4 forearm EMGs	92.7	67.9	94.2	81.3	77.2	87.5	83.5±10.0
1 best – all	78.1 (#7)	51.3 (#7)	76.2 (#7)	55.7 (#4)	61.0 (#7)	72.9 (#7)	65.9±11.4
1 best - hand	71.8 (#4)	47.1 (#3)	67.4 (#2)	55.7 (#4)	50.7 (#4)	65.4 (#1)	59.7±10.0
1 best - forearm	78.1 (#7)	51.3 (#7)	76.2 (#7)	55.6 (#7)	61.0 (#7)	72.9 (#7)	65.9±11.4
All 8 EMGs	97.5	81.8	97.1	92.4	82.0	91.5	90.4±7.0

Muscles: #1 opponens pollicis, #2 abductor pollicis brevis, #3first dorsal interrosseus, medial head, #4 first dorsal interrosseus, lateral head, #5 flexor carpi radialis, #6extensor digitorum, #7 extensor carpi ulnaris, #8 extensor carpi radialis.

**Table 2:** Reconstruction and recognition accuracy for different muscles.

Muscle	Individual muscles	Hand versus forearm	All muscles
	Mean±st. dev.	Mean±st. dev.	Mean±st. dev.
Reconstruction,	R^2^: X; Y		
Opponens pollicis	0.09±0.05; 0.33±0.15	Hand:	0.16±0.13; 0.25±0.10
Abductor pollicis brevis	0.11±0.05; 0.21±0.06	0.12±0.07; 0.27±0.10	
First dorsal interosseous (m)	0.13±0.08; 0.27±0.08		
First dorsal interosseous (l)	0.14±0.10; 0.26±0.08		
Flexor carpi radialis	0.15±0.16; 0.22±0.13	Forearm:	
Extensor digitorum	0.18±0.11; 0.20±0.06	0.21±0.16; 0.24±0.09	
Extensor carpi radialis	0.31±0.16; 0.27±0.11		
Extensor carpi ulnaris	0.20±0.19; 0.27±0.07		
Recognition,	% correct		
Opponens pollicis	49.4±13.3	Hand: 51.4±10.9	51.6±12.5
Abductor pollicis brevis	47.6±12.0		
First dorsal interosseous (m)	55.2±7.9		
First dorsal interosseous (l)	55.1±9.2		
Flexor carpi radialis	47.7±12.4	Forearm: 51.8±14.1	
Extensor digitorum	45.1±10.8		
Extensor carpi radialis	65.9±11.4		
Extensor carpi ulnaris	48.8±14.2		

In the EMG recognition approach, we used linear discriminant analysis [16] to translate EMG patterns into font characters. Figure 3A illustrates the operation of written-character discrimination algorithm. The subjects were asked to write characters, numbers from “0” to “9” (50 repetitions per characters). A half of the records (250 randomly selected trials) were used as the training set for the discriminant analysis, and the remainder of the records was used as a sample set. During the training phase, 3.5 s epochs corresponding to single characters were selected using an algorithm in which bursts in compound EMG (the sum of rectified EMGs from all muscles) were detected which crossed a threshold (0.5 standard deviation from the intertrial level) designated the epoch onset. Averaging the EMGs over all detected epochs yielded a generic template. This template was entered in the template matching analysis in which a 3.5 s sliding window was moved along the EMG records, and the correlation coefficient between the EMGs and the template was continuously correlated. Character writing epochs were then refined using the occurrences of peak correlations as epoch onsets. These epochs were then entered in the linear discriminant analysis that recognized the characters. The quality of recognition was evaluated across the 6 subjects using the percent of correct recognitions as the measure. This percent was 90.4±7.0 (mean±standard deviation across subjects). When hand muscle EMGs were analyzed separately (Table 1) the percent was 79.2±10.6, and for the forearm muscles we obtained the value 83.5±10.0. When a single best reconstructing muscle was selected, the percent of correct reconstructions was 65.9±11.4 (Table 1), and the average percent correct for any single muscle was 51.6±12.5 (Table 2).

**Figure 4:**
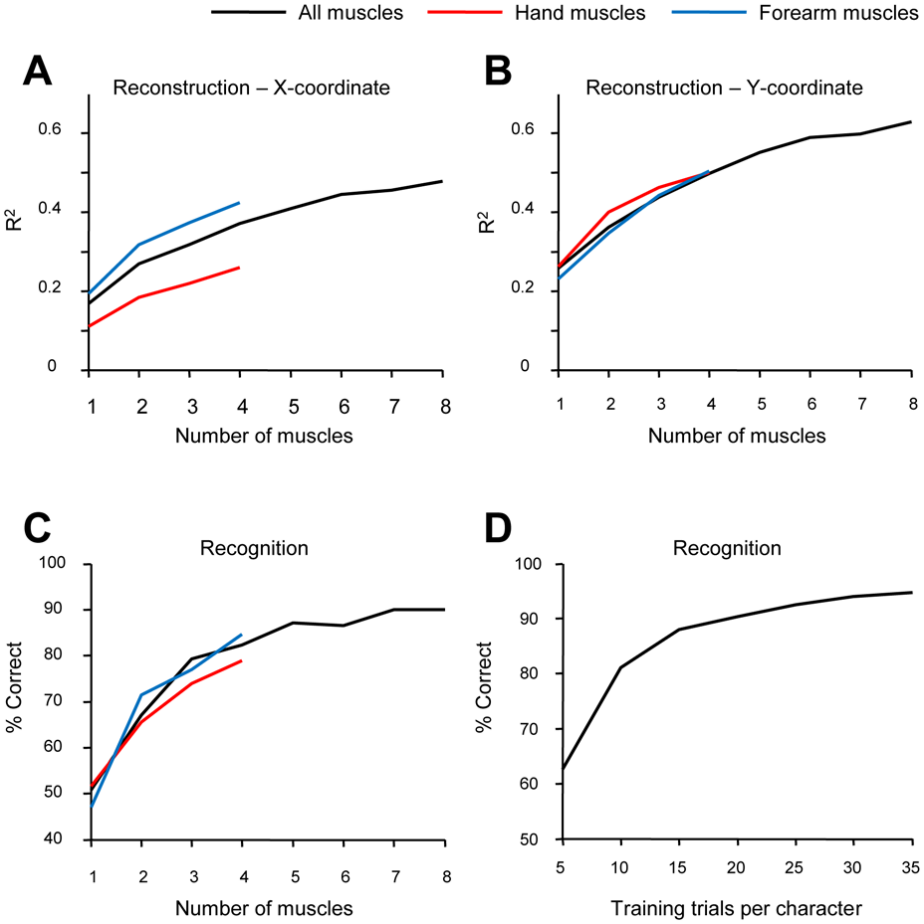
Performance of reconstruction and recognition algorithms as the function of the number of recorded EMGs and the amount of training data. The analyses were conducted were all muscles and hand and forearm muscles only (see key on top). A: Reconstruction accuracy of the X-coordinate of the pen as the function of number of muscles recorded. Muscles were taken in different combinations, and *R^2^* was averaged across these combinations and across subjects. B: Recognition accuracy of the Y-coordinate. C: Recognition accuracy as the function of the number of recorded muscles. D: Improvement in recognition accuracy as the function of training set size.

As shown in Fig. 4, the performance of both the reconstruction algorithm (Fig. 4A,B) and the recognition algorithm (Fig. 4C) benefited from the EMG recordings from multiple muscles. Further, we estimated the performance of discriminate analysis for different amounts of training data. In this analysis, different amounts of data were taken from the recordings as the training set, and the rest of the data was used as a sample set. Analysis of a representative experimental session is shown in Fig. 4D. A minimum of five repetitions per font character were needed for the discriminant analysis to work. Recognition accuracy was 63% correct for this amount of training data. As the number of repetitions increased to 35 per character, recognition improved to 97% correct.

## Discussion

Thus, we have shown that EMGs of hand and arm muscles can be converted into handwriting patterns: either the actual handwriting traces or font characters. This demonstration opens a number of directions for future research and practical applications. First, we have shown that EMG-based technology is a viable alternative to traditional methods of record taking. We envision a computer peripheral, an EMG glove, in which electrical activity of hand and/or forearm muscles are streamed directly to a computer where mathematical algorithms transform it into font characters. Such technology can employ dry electrode attachments and can be interfaced to the computer using wireless technology. As such, it may both offer certain advantages to conventional digitizing technologies, such as digitizing tablets, and become a useful supplement to these technologies. Our methodology can be also applied to clinical studies. While handwriting is impaired in dementia [17], Parkinson's disease [18], writing tremor [19], and attention deficit hyperactivity disorder [20], EMG changes during these handwriting impairments are poorly understood. Our techniques for extracting handwriting patterns from hand and forearm EMGs may contribute to both clinical research and development of clinical devices that would assist patients with impaired handwriting.

## Methods

### EMG and handwriting recordings

This study was approved by the Institutional Review Board of St. Lawrence University, Canton, NY. The data were recorded from 6 subjects. No personal information was recorded during sessions and all data were analyzed anonymously. Written informed consent was obtained from the subjects prior to the EMG recording sessions.

Each subject was comfortably seated at a desk in front of a computer monitor and wrote on a digitizing table with a pen while looking at the computer monitor that displayed the written traces. EMGs of 8 muscles were simultaneously recorded (Fig. 1a). Since the handwriting involves both the finger and wrist movements, surface EMGs were recorded from intrinsic hand and forearm muscles that produce these movements (Fig. 1b). Bipolar surface EMG electrodes were placed on four forearm muscles: flexor carpi radialis (FCR), extensor digitorum (ED), extensor carpi ulnaris (ECU), extensor carpi radialis (ECR) and four intrinsic hand muscles: opponens pollicis (OP), abductor pollicis brevis (APB), and medial (mFDI) and lateral (lFDI) heads of first dorsal interrosseus). The grounding electrode was placed on the subjects forehead. The skin surface overlying the muscles of interest was first cleaned with alcohol and the electrodes were prepped with electrode paste, firmly pressed to the skin, and fixed in place with hypoallergenic tape. After the electrodes were attached, the whole assembly was wrapped with an elastic bandage (Fig. 1a) to fix the electrode leads to minimize the occurrence of mechanical artifacts in the recordings.

Our system acquired data from three sources: EMG signals were obtained using a amplified recording system (Grass, model 15LT/15A54-2 quad modules) with a digitizer (Polyview/16SYS), pen tracking was accomplished using an Intuos 3/Wacom tablet, and pen contact was detected with pressure sensitive piezo film attached to the tablet. The tablet data were sampled at 100 Hz, which included mouse click events generated when the pen touched the tablet or was lifted from the tablet. EMG signals were differentially amplified (1000X gain), band-pass filtered (-6 dB cutoff points at 5 Hz and 500 Hz) and sampled at 1 kHz per channel. The pressure sensitive piezo film was placed on the writing surface of the tablet and the purpose of the film was simply to indicate the onset of the writing session by generating a triggering pulse (5 V, 0.05 s) sent to one channel of the EMG amplifier. EMG acquisition was handled by Grass software, and a MATLAB script controlled the acquisition of the data from the tablet.

Each subject was instructed to write numeric characters from “0” to “9”. The handwritten traces were displayed on the computer monitor mounted in front of the subject. The subjects were instructed to write the numbers within an 8×8 cm writing area on the digitizing tablet using their individual handwriting patterns. Each subject held the pen in the hand according to his/her individual habits. By a *trial* we define a recording epoch during which a subject wrote a single character. Each character was successively written 50 times. Therefore, each subject wrote 500 characters (i.e. performed 500 trials) during a daily recording session. Subjects could rest for a few minutes in between the recordings of individual characters, but the electrodes were not removed. Since in this study we sought to recognize individual characters, the subjects were asked to make pauses in between the characters. The trials were paced by the computer software which displayed a fresh writing area in the beginning of each trial. The duration of each trial was 7 s of which 2–3 s corresponded to character writing. Representative examples of the EMG signals and handwriting traces are shown in Fig. 3a.

### Data analysis

Handwriting patterns were extracted from rectified EMGs. Rectified EMGs were calculated by full-wave rectification of the original EMG signals followed by low-pass filtering with a cutoff frequency of 5 Hz (second order Butterworth filter).

In the handwriting *reconstruction* algorithm, we reconstructed pen traces from the EMGs of eight muscles of the arm. Thus, the end result of this method was the trace of the character with the only difference that it was not actually written by a pen, but rather derived from the EMGs (Fig. 2b). Handwriting traces were extracted from the EMGs using a linear method, the Wiener [14]. In the *recognition* algorithm, we recognized the characters written by the subjects by comparing the EMG patterns to a set of previously obtained EMGs. The recognition choice was the character whose previously recorded EMG patterns most closely matched the examined EMG pattern.

In both the reconstruction and recognition methods, the analysis consisted of two steps: (1) training the algorithm and (2) decoding using the trained algorithm. Accordingly, the experimental data (50 trials per each of 10 numerical characters) were split into two separate parts: (1) training data and (2) decoding data. To split the data into these parts, we simply used the first half of the record for each character for training and the second part for decoding. Thus, 250 trials (25 trials per character) were used for training and separate 250 trials were used for decoding (cross-validation) within one recording session for one subject.

To reconstruct handwriting into traces, we started with the application of a linear model that decoded pen coordinates *x(t)* and *y(t)* as a weighted linear combination of the EMG inputs:
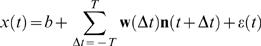
(1)where *x(t)* is X-coordinate at time 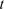, 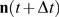 is a vector of input signals (rectified EMGs on 8 channels), at time 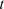 and time-shift 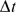 (negative shifts correspond to past values, positive shifts correspond to future values), *T* is the time window for the lags were, 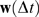 is a vector of weights for each input at time-lag 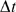, 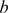 is the y-intercept, and 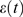 is the residual error.

Wiener filter defined by equation 1 reconstructs pen position from the EMG activity of several muscles sampled in the interval –T to T centered on the time point for which the reconstructed variable is calculated. EMG samples in this window are given different weights to optimally map time-varying EMG activity pen coordinates.

Equation 1 was solved using linear least squares regression (MATLAB routine *regress*). This equation can be recast in matrix form as
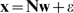
(2)where **x**, **w** and 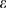 are column vectors, **N** is a matrix and *b* is a scalar. Rows in **x** and **N** correspond to time *t  = * {*t_start_*, *t_start_ + step*, *t_start_ + 2step*, …, *t_end_*}, and rows in **w** correspond to lags 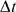  =  {*−T, −T + step, −T + 2step,* … *T*} and recording channels. In this notation, matrix **N** contains lagged data and thus has a column for each lag and each channel. The y-intercept is handled by prepending a column of ones to matrix **N**. The weights **w** are solved by
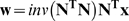
(3)where **N** and **x** are taken from model training datasets.

Linear model decoding for the y-coordinate of the pen was performed the same way as the decoding for the x-coordinate (equations 1–3).

For character recognition, we used Fischer linear discriminant analysis [16] to translate EMGs into the text characters represented by a computer (Fig. 3). To detect the onset of handwriting for each character, we used the compound EMG calculated as the sum of all analyzed rectified EMGs. Compound EMG was then detected as the threshold crossing set to 0.5 standard deviations from the intertribal level. After these onsets were determined, the EMG record was segmented into 3.5 s epochs which represented the writing of each character. Further, these epochs were downsampled to 10 samples per second (or 100 ms bins). A generic EMG template was calculated by averaging the EMG records across the epochs representing individual characters. This template consisted of the templates for individual muscles (35 bins per muscle) stacked together. Then, the template was slid across the EMG records, and correlation coefficient, *R*, between the EMGs and the template was calculated. *R* was high when the template was aligned with character writing episodes. Peak *R* values were then used to better segment the EMGs into 3.5 s epochs corresponding to each character: the onsets of these epochs were set to the occurrences of peak *R*. These segments were entered in the discriminant analysis (MATLAB function *classify*) as the training data. To reduce data dimensionality, principal component analysis was used to preprocess the EMG data before the discriminant analysis step. Empirically, the best results were obtained when the number of parameters was reduced from 280 (35 bins for each of eight muscles) to 50 principal components.
